# A Case Report of Simultaneous Intracranial Hemorrhage and Cerebral Venous Sinus Thrombosis in a Young Indian Male: Diagnostic and Therapeutic Challenges

**DOI:** 10.7759/cureus.55642

**Published:** 2024-03-06

**Authors:** Rahul S Patil, Ahsan A Faruqi

**Affiliations:** 1 General Medicine, Dr. D. Y. Patil Medical College, Hospital, and Research Centre, Pune, IND

**Keywords:** seizure, headache, intracranial hemorrhage, vegetarian diet, hyperhomocysteinemia, folic acid deficiency, vitamin b12 deficiency, thrombosis, cerebral venous sinus thrombosis (cvst), cvst

## Abstract

This case report discusses the intricate diagnostic and therapeutic challenges faced by a 23-year-old Indian male who presented with altered consciousness, a holo-cranial headache, right-sided hemiparesis, and subsequent neurological symptoms. The patient’s dietary habits, leading to vitamin B12 and folic acid deficiencies resulting in hyperhomocysteinemia, along with binge alcohol drinking leading to dehydration, were identified as the main causes of cerebral venous sinus thrombosis (CVST) in this case. The case was complicated by an additional cerebral hemorrhage. The patient received a comprehensive treatment regimen involving antiepileptic medications, intravenous fluids, and anticoagulation therapy. A decline in the Glasgow Coma Scale score prompted further interventions. Collaborative decision-making, involving neurologists, neurosurgeons, and the patient’s relatives, steered the treatment course, ultimately favoring continued medical management over decompression surgery. Notably, the patient exhibited remarkable progress in mobility, achieving the ability to walk with support by the end. This case report contributes valuable insights to the understanding of CVST, emphasizing the significance of nutritional considerations, especially in vegetarians, and underscoring the importance of thorough diagnostic evaluations in complex clinical scenarios.

## Introduction

Cerebral venous sinus thrombosis (CVST) refers to the occurrence of blood clot formation within the brain's venous sinuses, which obstructs normal blood outflow. This obstruction poses a risk of blood cell rupture, releasing blood into the brain tissue and resulting in hemorrhage. The estimated annual incidence of CVST is approximately five cases per one million individuals [1]. CVST predominantly affects women in the younger age groups, mostly attributed to its correlation with pregnancy, the postpartum period, and hormonal contraception [2]. Thrombus formation most commonly occurs in the superior sagittal, sigmoid, and transverse sinuses, with superior sagittal sinus occlusion closely linked to intracerebral bleeding [3].

The primary presenting complaint in CVST is typically a headache, reported in 70-90% of cases, often without concurrent neurological examination abnormalities. Headaches may manifest suddenly, resembling the severe onset observed in subarachnoid hemorrhages. Additional symptoms include altered sensorium, papilledema, convulsions, and focal neurological indications, although these are less prevalent. Because of the nonspecific nature of these symptoms, physicians should maintain a heightened suspicion of CVST, especially in cases with a new-onset and progressively intensifying headache [4,5].

This case report explores the clinical presentation of a young Indian male with a headache, seizures, and unilateral extremity weakness. Upon imaging, the patient was diagnosed with CVST, accompanied by an underlying intraparenchymal hemorrhage. The main identified risk factors after thorough evaluation were vitamin deficiencies leading to hyperhomocysteinemia and alcohol binges leading to dehydration. The discussion delves into the challenges and considerations of managing CVST with concomitant intraparenchymal hemorrhage, shedding light on the intricacies of this uncommon but critical clinical scenario.

## Case presentation

A 23-year-old Indian male was brought to our emergency department by his relatives in a state of altered consciousness. According to the information provided by the family, the patient began experiencing symptoms three days ago after consuming 190 grams of alcohol in a binge, starting with a dull holo-cranial headache that progressively intensified. Afterward, he developed weakness in the right upper and lower limbs, followed by a generalized tonic-clonic seizure (GTCS) this morning. Subsequently, he remains in an altered state.

Upon examination, the patient was afebrile, with a pulse rate of 84 beats per minute, blood pressure of 140/90 mmHg, and oxygen saturation of 94% in room air. Pupils were bilaterally reactive to light. The patient appeared drowsy and disoriented by time, place, and person. The Glasgow Coma Scale (GCS) was 11/15, with movement observed only on the left side of his body. Plantar reflexes indicate right-extensor and left-flexor responses.

**Figure 1:**
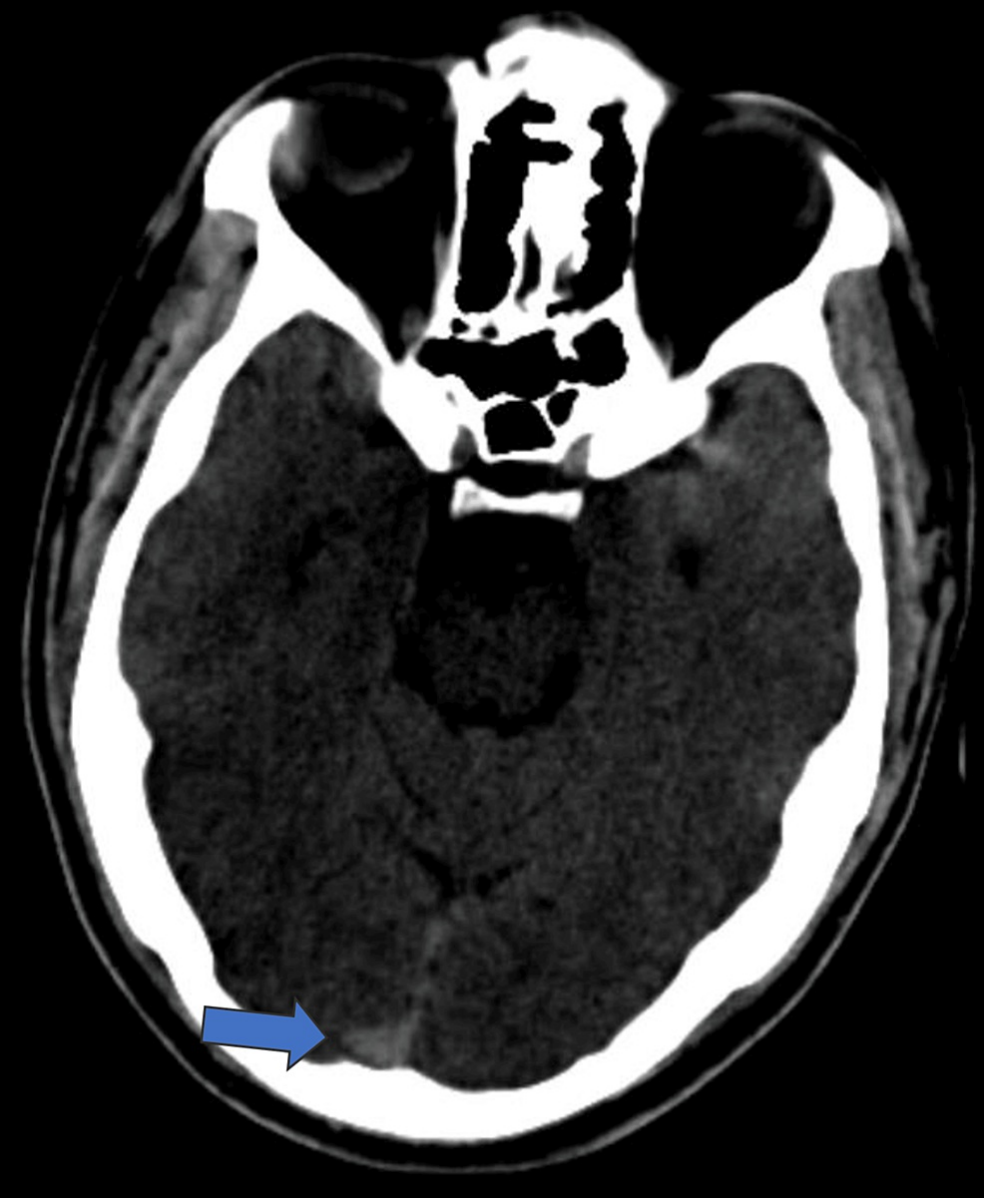
CT brain in axial view showing dense superior sagittal sinus (blue arrow) CT: computed tomography

**Figure 2:**
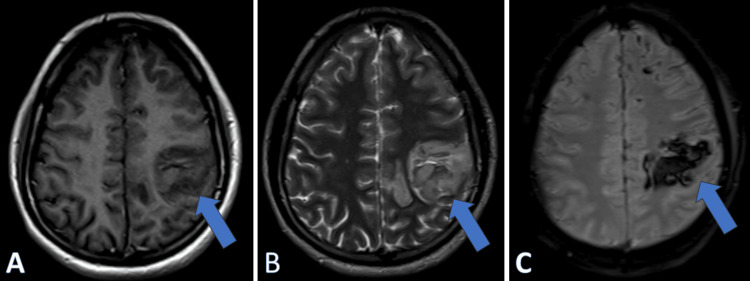
MRI brain in axial view reveals a well-defined area (blue arrows) of size 34 x 40 x 33 mm (CC X AP X TR) in the left frontoparietal region, showing heterogenous hyperintensity in T1 WI (A) and T2 WI (B) and blooming on GRE sequence (C) MRI: magnetic resonance imaging, CC: craniocaudal, AP: anteroposterior, TR: transverse, WI: weighted imaging, GRE: gradient echo recall

**Figure 3:**
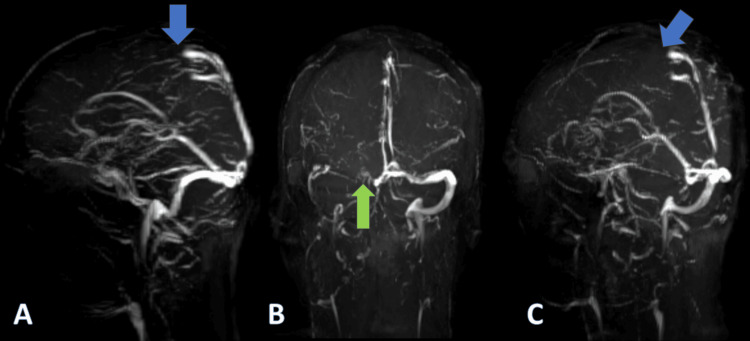
MRV in sagittal (A), coronal (B), and oblique (C) views showing loss of flow intensity in anterior two-thirds of the superior sagittal sinus (blue arrows) and reduced flow intensity in the left transverse and sigmoid sinus (green arrow) MRV: magnetic resonance venography

A preliminary diagnosis of cerebral stroke affecting the left hemisphere was considered. Additionally, there was a suspicion that CVST contributed to the headache. A bedside fundoscopy revealed no signs of papilledema. An initial CT of the brain showed a dense superior sagittal sinus, raising suspicion for CVST (Figure 1). To obtain a more detailed picture of the scenario, a brain MRI scan was performed, which revealed hemorrhagic infarction in the left frontoparietal region (Figure 2A-2C) and CVST (Figure 3A-3C). Laboratory investigations on admission (Table 1) showed elevated homocysteine and hematocrit levels, along with low vitamin B12 and folic acid levels. Additional tests (Table 2) were ordered to rule out other causes of thrombosis, which turned out to be negative, making hyperhomocysteinemia and dehydration post-alcohol binge the main causes in this case.

**Table 1:** Laboratory investigations done on admission SGOT: serum glutamic-oxaloacetic transaminase, SGPT: serum glutamic pyruvic transaminase, INR: international normalized ratio, aPTT: activated partial thromboplastin time, hs-CRP: high-sensitivity C-reactive protein, ESR: erythrocyte sedimentation rate, HIV: human immunodeficiency, HBsAg: hepatitis B Antigen, HCV-Ab: hepatitis C antibody

Parameters	Report	Normal limit
Hemoglobin	13 gm/dl	13.2-16.6 gm/dl
Mean corpuscular volume	100 fL	78.2-97.9 fL
Hematocrit	52.02%	38.30-48.60%
Total leucocyte count	22500 /µL	4,000-10,000 /L
Platelet count	1,07,000 /µL	1,50,000-4,10,000 /µL
Serum vitamin B12	<83 pg/ml	187-883 pg/ml
Serum folic acid	1.20 ng/ml	3.10-20.50 ng/ml
Serum homocysteine	56.27 µmol/L	5.08 to 15.39 µmol/L
Serum urea	28 mg/dL	17-49 mg/dL
Serum creatinine	0.77 mg/dL	0.6-1.35 mg/dL
SGOT	42 IU/L	8-48 IU/L
SGPT	25 IU/L	7-55 IU/L
Serum bilirubin	0.50 mg/dL	0.2-1.2 mg/dL
Serum albumin	4.1 gm/dl	3.4-4.8 gm/dL
INR	1.02	0.85-1.15
aPTT	25.76 secs	21.76-32.54 secs
D-dimer	300 ng/ml	0 to 500 ng/ml
hs-CRP	98 mg/L	up to 5 mg/L
ESR	25 mm/hr	up to 20 mm/hr
HIV/HBsAg/HCV-Ab	Non-reactive	Non-reactive
Random blood sugar level	96 mg/dl	up to 140 mg/dl

**Table 2:** Additional investigations Ig: immunoglobulin, ANA: antinuclear antibody, IF: immunofluorescence

Parameters	Report	Normal limit
Cardiolipin antibodies IgA	0.3	0.0-0.8
Cardiolipin antibodies IgG	0.2	0.0-0.8
Cardiolipin antibodies IgM	0.4	0.0-0.8
Beta 2 glycoprotein IgM	5.26 U/ml	0.0-12.0 U/ml
Beta 2 glycoprotein IgG	7.14 U/ml	0.0-12.0 U/ml
Lupus anticoagulant	26.5 secs	25.78-32.58 secs
Antiphospholipid antibody IgM	8.97 U/ml	0.0-12.0 U/ml
Antiphospholipid antibody IgG	4.20	0.0-12.0 U/ml
Protein C	90 IU/dL	65-135 IU/dL
Protein S	88 IU/dL	60-160 IU/dL
Antithrombin III	82%	75-125%
ANA by IF	Negative	Negative

A loading dose of injection (Inj) levetiracetam 1 gram intravenous (IV) stat, followed by 500 mg IV twice daily (BD) Inj, low-molecular-weight heparin (LMWH) 60 mg IV BD, and IV fluid resuscitation, was commenced. Subsequently, the patient was transferred to the intensive care unit for continuous monitoring. The following day, the patient's GCS declined to 8/15 with two additional episodes of GTCS, effectively managed by Inj lorazepam 4 mg IV stat doses. The dosage of levetiracetam was increased to 1 gram IV BD.

**Figure 4:**
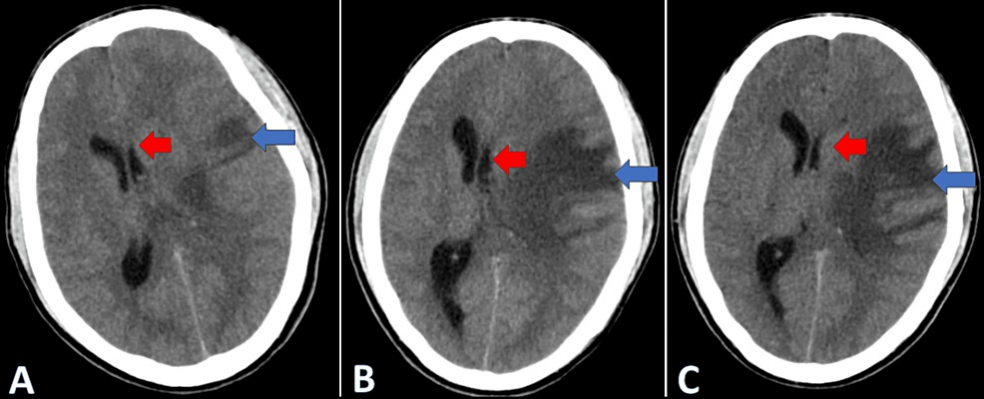
Serial CT brains (axial views) done on the third (A), seventh (B), and 10th (C) day shows the mass effect on the left lateral ventricle and a midline shift to the right side (red arrow) and effacement of surrounding sulci and gyri (blue arrow) due to cerebral edema CT: computed tomography

In response to the drop in GCS, a CT brain scan was performed, revealing an enlargement of the hemorrhagic lesion with a midline shift (Figure 4A). The patient's condition continued to deteriorate, reaching a GCS of 7/15 the following day. A repeat CT brain scan showed an increase in cerebral edema (Figure 4B-4C), prompting a neurosurgery referral. After collaborative discussions with the neurologist, neurosurgeon, and the patient's relatives expressing reluctance toward decompression surgery, a decision was made to continue with medical management. The patient received acetazolamide and antiepileptics, and the observation persisted.

**Figure 5:**
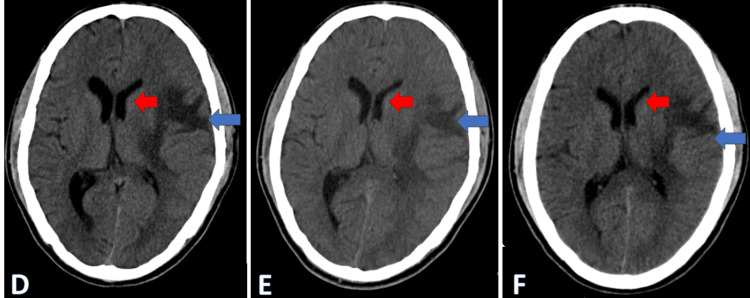
Serial CT brains (axial view) done on Day 13 (D), Day 21 (E), and Day 28 (F) showing a decrease in mass effect on the left ventricle (red arrow) and cerebral edema (blue arrow) CT: computed tomography

**Figure 6:**
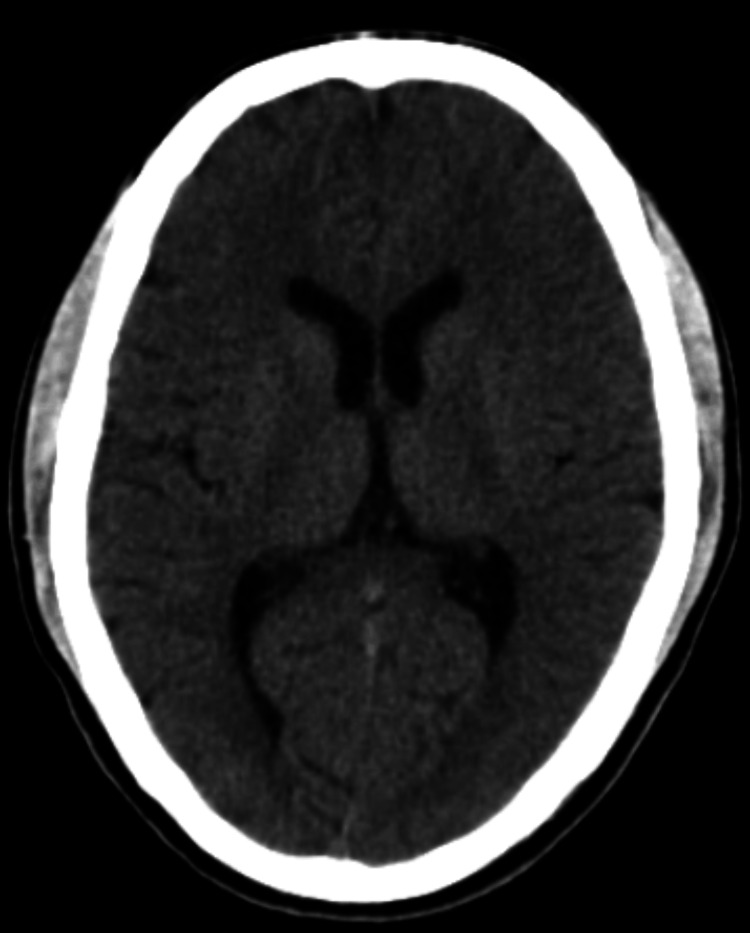
Follow-up CT brain (axial view) done one month after discharge CT: computed tomography

Supportive care was sustained, leading to the patient regaining consciousness on day 12 with an improved GCS of 15/15. Inj LMWH was discontinued after a total of 10 days, and the patient was transitioned to the oral anticoagulant rivaroxaban 20 mg once daily. A follow-up CT brain scan of the patient showed a decrease in midline shift and cerebral edema (Figure 5D-5F). Subsequently, the patient was moved to the ward, where daily physiotherapy was commenced. Remarkably, after 25 days of physiotherapy, the patient achieved the ability to walk with support and was discharged after a 40-day hospital stay. Subsequent follow-ups revealed an uneventful recovery, with noticeable improvement in right-sided weakness clinically and the absence of midline shift and cerebral edema radiologically (Figure 6).

## Discussion

**Figure 7:**
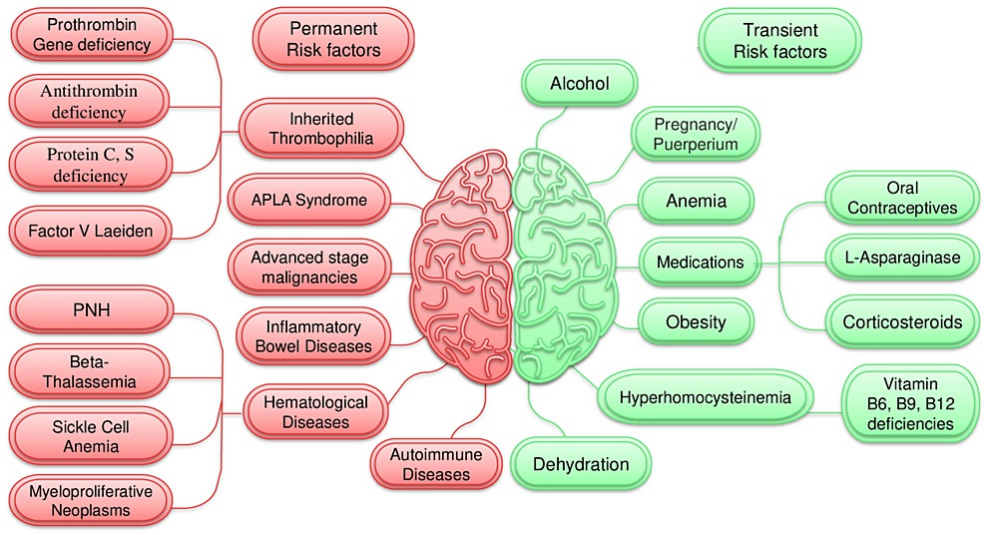
Permanent and transient risk factors of CVST CVST: cerebral venous sinus thrombosis, PNH: paroxysmal nocturnal hemoglobinuria, APLA: antiphospholipid antibody syndrome Rounded red rectangles: permanent risk factors, rounded green rectangles: transient risk factors Image Credit: Dr. Ahsan A. Faruqi

The etiology of CVST is intricate, involving a myriad of both non-reversible and reversible risk factors (Figure 7). At least one risk factor is found in 85% of patients, and the combination of several risk factors is responsible for 50% of occurrences. A small percentage of cases are still idiopathic [4,6].

Identifying CVST and concurrent intracerebral hemorrhages presents a formidable challenge in clinical practice. This complexity arises due to several factors. Initially, the symptoms of CVST can be nonspecific, often overlapping with other neurological conditions. The hallmark symptom, headache, may be present in a myriad of neurological disorders, making it challenging to pinpoint CVST solely based on clinical presentation. Moreover, the simultaneous occurrence of intracerebral hemorrhage compounds the diagnostic challenge. Hemorrhagic events can mask the typical signs of CVST or be erroneously attributed solely to bleeding, leading to potential delays in diagnosis [7]. In their study, Ferro et al. observed a median delay of seven days from the onset of symptoms to the diagnosis of CVST. Furthermore, the study concluded that in isolated intracranial hypertension syndrome patients, a diagnostic delay was significantly associated with adverse outcomes such as death or dependency [8].

The pathogenesis of CVST remains not entirely known, primarily due to the considerable variability of the venous system. Nonetheless, two key mechanisms are postulated to contribute to the clinical manifestation of CVST. Firstly, the thrombosis of cerebral veins impedes the normal drainage of blood from brain tissue, precipitating an elevation in venous and capillary pressure. This, in turn, disrupts the blood-brain barrier, resulting in vasogenic edema. The compromised barrier integrity leads to the leakage of blood plasma into the interstitial space. The escalating pressures within the venous system perpetuate a continuous increase in intracranial pressure, fostering localized cerebral edema. Furthermore, the heightened pressure may result in intraparenchymal hemorrhage, attributed to the rupture of veins or capillaries. The second implicated mechanism involves the obstruction of the venous sinus, leading to diminished cerebrospinal fluid absorption and subsequent elevation in intracranial pressure [9].

The elevation in intracranial pressure, stemming from reduced cerebrospinal fluid reabsorption, forms the pathophysiological basis for headaches in CVST. Notably, the Valsalva technique and the supine position often exacerbate headaches, highlighting the connection between intracranial pressure dynamics and symptom manifestation. Approximately one-quarter of CVST patients experience focal seizures, whereas another quarter initially present with focal seizures that subsequently generalize. The remaining half experienced generalized seizures from the onset. In comparison to arterial stroke cases, patients with CVST demonstrate a higher propensity for seizures, which can be potentially attributed to the accumulation of catabolic products resulting from venous stagnation [10]. Notably, individuals exhibiting focal deficits are more likely to have superficial system thrombosis involving the parasagittal cortex, which encompasses critical motor and sensory regions [4]. Our patient presented with a headache, altered sensorium, and focal seizures, which later became generalized.

Elevated homocysteine levels, referred to as hyperhomocysteinemia, represent a physiological state arising from heightened homocysteine levels-an amino acid derived from methionine with sulfhydryl properties that can induce toxicity to neurons and the vascular endothelium [11]. Crucial cofactors in the homocysteine metabolism pathways, namely folic acid, vitamin B12, and pyridoxine, play pivotal roles, and their deficiencies can result in hyperhomocysteinemia. This condition can trigger oxidative stress, endothelial dysfunction, and a prothrombotic state. A study conducted in Northern India by Kalita et al., involving 97 patients, revealed that 52% of individuals with CVST exhibited hyperhomocysteinemia, underscoring the significance of dietary deficiencies, particularly vitamin B12, as a risk factor in the development of CVST [6]. Notably, our patient, being a vegetarian, presents a significant aspect contributing to the observed deficiencies in vitamin B12 and folic acid. In this context, it emerges as one of the main identifiable causes for the development of CVST in this case.

In a case series published by Sohoni et al., two male patients aged 40 and 41 developed CVST after a binge of alcohol [12]. The present case shares similarities with these two cases in terms of presentation and a history of an alcohol binge. However, it differs by exhibiting high serum homocysteine levels and low vitamin B12 and folic acid levels [12]. While heavy alcohol consumption raises the relative risk of stroke, a meta-analysis of 35 studies suggests that moderate drinking may lower the overall risk of stroke [13].

Laboratory examinations and neuroimaging are pivotal in the diagnosis of CVST. D-dimer levels serve as a helpful tool in identifying patients with a low probability of CVST. However, it is crucial to emphasize that a normal D-dimer level, as indicated by these assessments, should not lead to the dismissal of further evaluation, especially in cases with a substantial clinical suspicion of CVST. Although a standard CT or MRI scan is valuable in the initial assessment of suspected CVST, a negative result does not conclusively rule out the condition. In such scenarios, a venographic study, be it magnetic resonance venography or CT venography, becomes imperative to either confirm or outline the extent of CVST [14].

Evidence-based recommendations from the American Heart Association outline the treatment approach for CVST. These recommendations include the early commencement of antiepileptic drugs for patients with a single seizure, with or without parenchymal lesions. In the absence of seizures, routine use of antiepileptic medication is not advised. Initial anticoagulation with low molecular weight or unfractionated heparin, followed by the use of vitamin K antagonists, is considered reasonable. Acetazolamide is viewed as an acceptable treatment for elevated intracranial pressure, and decompressive hemicraniectomy is a viable option in cases of significant cerebral bleeding or mass effect leading to uncontrollably high intracranial pressure [14]. In this specific case, the patient underwent a comprehensive management approach that included antiepileptic medications, acetazolamide, and anticoagulation. Despite the potential indication for decompressive surgery, a noteworthy aspect is the influence of the patient's relatives, who were hesitant about opting for such a procedure. Despite this challenge, a collaborative decision was reached to continue with ongoing medical management, complemented by vigilant monitoring. This collective approach aimed to navigate through the intricacies of the case while respecting the preferences of the patient's family.

## Conclusions

This case report provides valuable insights into the management of a complex scenario involving a 23-year-old Indian male diagnosed with CVST and intraparenchymal hemorrhage. The patient's vegetative dietary practices, which led to deficiencies in vitamin B12 and folic acid, combined with binge alcohol consumption and dehydration, emerged as significant risk factors. The chosen management approach, which included antiepileptic medications, acetazolamide, and anticoagulation, prioritized continued medical management over decompressive surgery. This decision was guided by collaborative discussions involving the patient's relatives.

The key takeaway underscores the importance of a tailored and multidisciplinary approach when dealing with the challenges posed by CVST cases. This involves maintaining a high index of suspicion for CVST, especially in cases with nonspecific symptoms such as headaches and seizures. Thorough diagnostic evaluations, encompassing neuroimaging and laboratory tests, play a crucial role in facilitating early intervention. Additionally, the case highlights the necessity of considering nutritional factors, emphasizing the importance of supplementing vegetarians with vitamin B12. Ultimately, clinicians can draw on these lessons to navigate similar complex clinical scenarios involving CVST, taking into account multiple contributing factors and engaging in collaborative decision-making.
